# The intergenerational effects of war on the health of children

**DOI:** 10.1186/1741-7015-12-57

**Published:** 2014-04-02

**Authors:** Delan Devakumar, Marion Birch, David Osrin, Egbert Sondorp, Jonathan CK Wells

**Affiliations:** 1 Institute for Global Health, University College London, London, UK; 2 Medact, London, UK; 3 Royal Tropical Institute, Amsterdam, Netherlands; 4 Childhood Nutrition Research Centre, Institute of Child Health, University College London, London, UK

**Keywords:** War, Conflict, Developmental origins, Children, Mental health

## Abstract

**Background:**

The short- and medium-term effects of conflict on population health are reasonably well documented. Less considered are its consequences across generations and potential harms to the health of children yet to be born.

**Discussion:**

Looking first at the nature and effects of exposures during conflict, and then at the potential routes through which harm may propagate within families, we consider the intergenerational effects of four features of conflict: violence, challenges to mental health, infection and malnutrition. Conflict-driven harms are transmitted through a complex permissive environment that includes biological, cultural and economic factors, and feedback loops between sources of harm and weaknesses in individual and societal resilience to them. We discuss the multiplicative effects of ongoing conflict when hostilities are prolonged.

**Summary:**

We summarize many instances in which the effects of war can propagate across generations. We hope that the evidence laid out in the article will stimulate research and – more importantly – contribute to the discussion of the costs of war; particularly in the longer-term in post-conflict situations in which interventions need to be sustained and adapted over many years.

## Background

**Figure 1:**
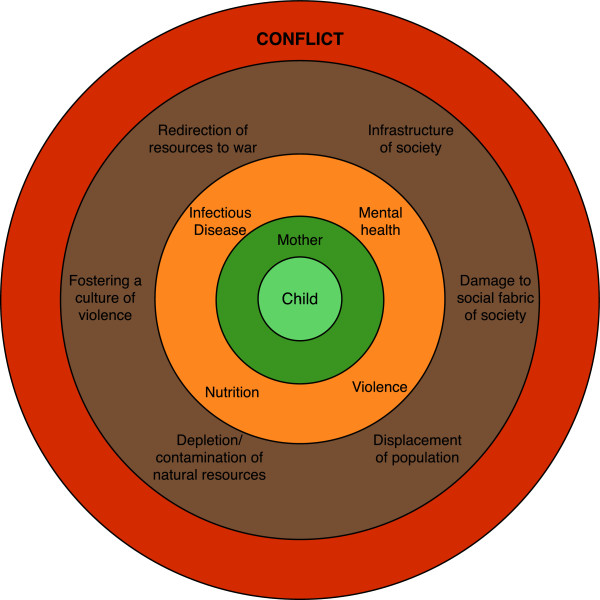
**Direct effects (orange) and indirect effects- as described by Levy **[5]** - (brown) of conflict on mother and child.**

The adverse effects of war on the health of children have been well documented [1-4], but less well known is how exposure to violence can propagate effects across generations. Conflict causes injury, illness and breakdown in the structures that provide preventive, curative and ameliorative care. It has profound effects on society that form a permissive framework for the effects we describe. The mediators of loss are many, but include population displacement and breakdown of health services and schooling, on a background of economic decline and supply constraint. Figure 1 shows how these indirect effects are related with conflict and have a pervasive influence that reaches down to the next generation.

Conflicts or wars, as defined by the World Health Organization^a^, are becoming increasingly complex and involve multiple state and non-state actors [6]. In the last 20 years, conflicts have occurred in 37% of countries. In 2010, there were 30 armed conflicts involving at least one state, 26 non-state conflicts and 18 armed groups involved in one-sided violence [7,8]. It is difficult to know if the nature of conflict has changed, but recent wars provide evidence of the targeting of health facilities to weaken opposition forces and populations. Even schools are coming under attack, either deliberately or as collateral damage [9,10]. The association between conflict and poverty means that conflict-affected populations face major challenges after fighting has ceased. Economic development is affected by war in a number of ways that involve a combination of reduced output and increased expenditure, for example on security, and weakened systems of governance [11]. Most modern wars are short-lived and occur within national boundaries, but some, such as recent conflicts in South Sudan and Afghanistan, continue for decades [6]. Economic breakdown within conflict-afflicted states makes some countries among the poorest in the world, with approximately a third being defined as low-income (gross national income per capita <US$995). The World Bank judges that 80% of the world’s 20 poorest countries have experienced major conflicts [12], and it has been estimated that conflict leads to a reduction in annual economic output of 1% to 3% [13,14].

**Figure 2:**
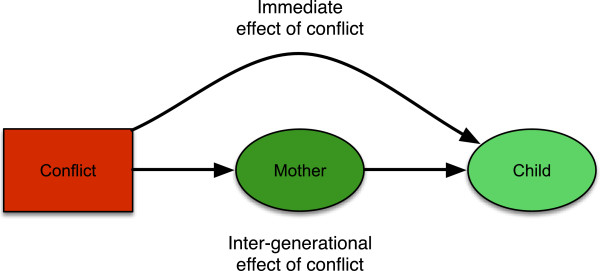
Immediate and intergenerational pathways through which conflict affects health.

That harms to health may be long-lasting within an individual’s lifetime is well established, but there is increasing awareness that adverse effects may continue through intergenerational biological mechanisms. Our life course is sensitive to the environments in which we, our mothers, and grandmothers were conceived and grew up. Many exposures during development are mediated by maternal phenotype and reflect stresses to which mothers were originally exposed. Figure 2 illustrates how the effects of conflict can be mediated through harm to mothers. Recent work has emphasized that maternal physiology and behavior can buffer their offspring against ecological stresses, but this is only partial [15], such that exposure to conflict in one generation may potentially propagate adverse effects to subsequent generations. Epigenetic modifications to DNA expression have emerged as key biological mechanisms contributing to such intergenerational transmission, although direct transmission of epigenetic marks themselves appears rare and the primary impact of maternal phenotype is its influence on *de novo* marks in the offspring [16,17].

Greater awareness of the potential intergenerational consequences of conflict may lead to their early recognition and improved diagnosis and response. Documentation of the consequences will also add to the overall evidence on the effects of conflict on health, which may weigh more heavily in the balance when violent conflict is an option. A war may end, but its effects do not, and – in an example of intergenerational justice - those who resort to conflict may be held accountable for harms to the health of future generations. For this review, we searched the Medline, Psychinfo and Google Scholar databases using the search strategy described in Additional file 1. Only articles published in English were included, with no date restrictions. After finding an article, we considered the reference list or suggested articles for further evidence. This was supplemented by a general search of the literature on conflict/war and specific searches as questions arose. We summarize the evidence of how four features of conflict - violence, challenges to mental health, infections and malnutrition - may harm more than one generation, responding to emerging ideas about the epigenetic transmission of physiology [18,19]. We also discuss the multiplicative effects of ongoing conflict when hostilities are prolonged.

### Violence

#### Maternal exposure

Violence is ubiquitous in war and pervades all strata of society, although its occurrence is probably underestimated [20-22]. The 20th century saw a secular trend toward a greater proportion of civilians affected, especially women and children [23]. It is estimated that up to 90% of war-related deaths in the last decade were civilian [5]. Conflict creates an ecosystem that persists, but is maladapted to peacetime, and its legacy is frequently an environment that fosters violence. Numbers of small arms remain for years, an example being the 1996 civil war in Guatemala, where rates of gun violence were greater after the war ended [24]. Similarly, increasing armed conflict in land disputes in East Africa is a consequence of prior cold war politics [25], and the 110 million landmines currently thought to remain in 68 countries [26] may lie dormant and harm civilians, both physically and mentally, decades after the original conflict [27,28].

Women are at particular risk of combatant and civilian injury, and of interpersonal violence. Violence against women increases in times of conflict through targeted acts, such as rape and domestic or intimate-partner violence. Sexual violence, including rape, assault, trafficking and prostitution, increases during many conflicts due to the breakdown of traditional safety structures [29]. It may also be used as an intentional strategy of domination, as reported in the former Yugoslavia and Rwanda [20,30]. The magnitude of sexual violence in conflict zones is difficult to quantify and tends to be under-reported [31]. Approximately half of a sample of women interviewed in Liberia had suffered sexual violence [21] and studies in East Timor and Kosovo showed increases to 23% and 15%, respectively, during conflict, compared to 10% and 2% in the post-conflict period [32,33]. A 2006 study from Lebanon suggested that domestic violence increased in areas where there was collective violence [22]. There are other, more complex, links. Substance use is associated with violence against women in non-conflict settings [34-36], although the evidence that it increases during conflict is limited. Clearer is the bidirectional association between violence and alcohol use [37-39].

#### Intergenerational associations

A clear example of an intergenerational effect is stillbirth after physical violence against a pregnant woman [40,41]. During the siege of Sarajevo, perinatal mortality and morbidity more than doubled, and there was a rise in congenital malformations from 0.4% to 3% [42]. Violence is also associated with an increase in premature birth [43], as was seen during the war in Croatia in the early 1990s [44]. Similar to this in non-conflict settings, a meta-analysis on abuse during pregnancy showed an increased odds ratio for low birth weight of 1.4 (95% confidence interval (CI) 1.1 to 1.8) [45].

Sexual violence has both short- and long-term effects. In the short-term, it is associated with an increase in sexually transmitted diseases, the intergenerational effects of which are discussed later. Maman and colleagues relate HIV infection to violence through three mechanisms: ‘forced or coercive sexual intercourse with an infected partner; by limiting women’s ability to negotiate safe sexual behaviors; and finally by establishing a pattern of sexual risk taking among individuals assaulted in childhood and adolescence’ [46]. Even when services are available, fear of violence may lead to a lower likelihood of accessing treatments to prevent mother-to-child transmission of HIV [47]. In the longer-term, sexual violence may affect parenting capacity. In Rwanda and Darfur, children born after rape have been shown to be at increased risk of neglect, abuse, malnutrition and abandonment [48]. Evidence from hospitals in South Kivu, Democratic Republic of Congo, suggests that when children are born from rape, their mothers are five times more likely to suffer isolation from the community (odds ratio (OR) 4.84; 95% CI 1.41 to 19.53) [49]. Children may also be victims of sexual violence [9], which may change its characteristics in times of conflict: a greater likelihood of gang rape was seen, for example, during conflict in the Democratic Republic of Congo. Sexual violence against children has profound effects on their mental and physical development, with implications for future pregnancies and relationships [50]. Mental illness, affecting a family’s ability to function, can persist long after conflict has ended: symptom rates in those who had suffered from mass violence remained elevated for a decade after the Cambodian conflict [51]. More generally, exposure to violence has been associated with long-term effects on children’s health, brain structure and neural function [52]. A violent environment may also have long-term effects on the maturational schedule. In Colombia, the secular decline in age at menarche accelerated during periods with high homicide rates, suggesting that psychosocial cues can alter the developmental trajectory [53].

Violence can affect family life, both economically and socially. Among Cambodian families in which one member was injured by a landmine, 61% were pushed into debt [54]. A study from Nicaragua found that women who suffered from abuse had 44% lower earnings [55]. Combat, death and displacement can also lead to the breakdown of family structures. Family members may shift roles, as was noted among women in Nepal who became combatants and replaced those lost during the conflict [56].

It is generally thought that experiencing violence as a child is a risk factor for committing child abuse as an adult [57]. There is some evidence that children who witnessed frightening events (for example, in the Spanish civil war of 1936) are ‘more immune to the horrors of violence’ [4]. This is especially so for child soldiers, who may experience dehumanizing conditions during a formative period, but there is a danger that maladaptive behaviors are passed on to the next generation. There are few long-term studies in this group. In a 16-year follow-up of child soldiers in Mozambique, men still suffered distress from their traumatic events, but there was no evidence that abusive relationships were passed on to their children [58].

Chemical or radioactive weapons can also damage future generations. While evidence of genetic effects of radiation on the children of survivors of atomic bombs is lacking, follow up studies have found evidence of severe learning difficulties and microcephaly in people exposed *in utero. In utero* exposure was also associated with decreased school performance and intelligence quotient (IQ) [59,60]. Case–control studies suggest that exposure of men to mustard gas in Iran and mineral contamination (particularly depleted uranium) in Iraq was associated with congenital abnormalities in their children [61,62].

### Mental health

#### *Maternal exposure*

Mental health conditions are common during conflicts, and are augmented by the breakdown of mental health services and community coping strategies, and by increases in stress levels and drug and alcohol use [63]. The most common conditions are depression, anxiety and psychosomatic disorders, but the most widely studied in relation to conflict is post-traumatic stress disorder (PTSD). PTSD is common among combatants, and child soldiers are known to suffer from a much higher than average incidence of psychological disturbances and mental illness [64-67]. PTSD is also found in civilian populations in both women and children [68,69]. De Jong *et al*. showed that PTSD and anxiety disorder were the most common mental illnesses in all populations, and exposure to violence increased their likelihood in the four conflict zones of Algeria, Cambodia, Ethiopia and Palestine [70]. In Kabul, mothers of children under five were found to have an increase in prevalence of PTSD from 10% to 53% if they experienced at least one armed conflict event [71], and women living in conflict areas in Bosnia and Herzegovina showed a sevenfold increase over a control group in PTSD prevalence [72]. Importantly, mental health disorders also rise in those not directly exposed to violence. Women are generally affected more than men and strong associations exist between the mental health of mothers and their children [63]. For example, in Algeria poor quality of camp housing and the general difficulties of daily living were both associated with increased odds of PTSD (OR 1.8, 95% CI 1.3 to 2.5 and 1.6, 95% CI 1.1 to 2.4, respectively) [73]. Mental illness is also common in children. A systematic review in a long-term conflict in the Middle East showed that children do suffer from a wide range of mental disorders, such as PTSD, depression, anxiety, functional impairment, behavioral problems and Attention Deficit Hyperactivity Disorder [74].

The evidence on drug use in conflict is partial. Drug use increases in groups that have been displaced, especially among combatants and former combatants, for which it may be a way of coping with traumatic situations or memories [75-78]. Economic pressures and increased availability can lead to a rise or change in drug use. For example, the conflict and related drug policy in Afghanistan have been linked to an increase in intravenous opiate use [79,80]. The pattern of illicit drug use depends on the setting: opiates are more common in Pakistan and Afghanistan, while benzodiazepines are common in Bosnia-Herzegovina [81]. A study of khat (a substance that contains a psychoactive compound similar to amphetamines) in north-west Somalia showed that recent use among ex-combatants was 60%, compared with 28% in civilian war survivors and 18% in civilians with no experience of war. Psychotic symptoms were linked with age of onset of khat use and binge consumption. Rates of severe disability due to mental disorders (primarily psychosis) in males over the age of 12 increased in relation to their experience of war, with a prevalence of 16% in ex-combatants, 8% in civilian war survivors and only 3% in those with no experience of war [82].

#### *Intergenerational associations*

While evidence is currently limited, parental trauma and psychosocial stress during conflict have been associated with adverse health effects in offspring, through both biological pathways (such as neuroendocrine and immune system modulation) and propagation of stressful social environments [83-85]. Stress may be a mediator through which parents transmit adverse effects to their children, in both conflict and non-conflict settings. In a large population-based cohort study, maternal stress during pregnancy was associated with a number of outcomes in children, including risk of infection and mental disorders [86]. Stress arising from childhood separation from parents during World War II was linked to long-term impairment in offspring social mobility and socio-economic position [87]. A study in people living near Gaza showed a strong association between maternal symptoms of depression, anxiety and PTSD, and PTSD symptoms in children. Avoidance behaviors in children were also associated with the degree of trauma exposure in the mother [88], similar to associations shown in animal studies [89]. Possible mechanisms include epigenetic changes in the hypothalamic-pituitary-adrenal axis (see Box 1) [90,91]. There is also some evidence that paternal PTSD can likewise lead to symptoms in children [92].

Stressful emotions can be passed from one generation to the next, but maternal buffering appears to provide an important damping effect, so that children are less affected than their parents. Children exhibit a form of resilience [93] that is related in turn to their mothers’ wellbeing, quality of caregiving and environmental support [94], but traumatic events, such as those occurring in a conflict, may override this buffering. For example, children of Australian veterans of the Vietnam war were found to have a suicide rate three times that of the general community [95] and elevated rates of PTSD and other mental illness have been described in the offspring of Holocaust survivors [96-100]. Mothers, but not fathers, who were themselves offspring of Holocaust survivors had ‘higher levels of psychological stress and less positive parenting’, while in the grand-offspring generation, children had less positive self-perception and were said by their peers to show ‘inferior emotional, instrumental, and social functioning’ [101].

There is also evidence of intergenerational mental health effects in non-conflict settings, and these are likely to be exacerbated by conflict. A meta-analysis by Surkan *et al*. showed that maternal depression or depressive symptoms were associated with an OR of 1.5 (95% CI 1.2 to 1.8) for children being underweight and 1.4 (95% CI 1.2 to 1.7) for stunting. The reasons for this include poorer antenatal care, increased risk-taking behaviors and impaired maternal caring [102], with reduced or early cessation of breastfeeding as one possible mediator [103,104]. Maternal mental illness has also been linked to children’s cognitive abilities [105]. In India, maternal common mental disorder was associated negatively with mental development in infants at six months of age [106] and, in Barbados, with cognitive outcomes [107]. The latter association persisted when assessed via a school entrance exam at 11 to 12 years of age [108].

There are several mechanisms whereby drug use may exert adverse intergenerational effects. Drug use by pregnant women can have transplacental effects or cause maternal ill-health or altered behavior. The manifestations may be acute - neonatal abstinence syndrome from opiate withdrawal, for example [109] – or lead to longer-term behavioral and cognitive changes [110]. Both drugs and alcohol are closely associated with mental illness in the user, which, in turn, can have detrimental effects on parenting ability and employment. The use of khat has been linked to mental illness, as well as affecting the reproductive system and being teratogenic [111]. However, the magnitude of the intergenerational effect of drug use associated with conflict remains to be established.

### Infectious diseases and health systems

#### *Maternal exposure*

Mortality in women and children during conflicts is predominantly the result of conditions related indirectly to violence: infectious disease, malnutrition and complications of pregnancy [8]. With the exception of a few examples for which limitation of movement has appeared to prevent transmission [112], higher rates of infectious diseases, such as shigellosis and cholera, are common in conflict zones [113,114]. As described in Table 1, the major drivers are movement of people, infrastructural deterioration, and breakdown in prevention and treatment [115]. Migration of people, for example, both increases infectious disease burden and exposes unimmunized populations to new pathogens.

**Table 1:** Common reasons for an increase in infectious diseases during conflict

**Causes of an increase in infectious disease**	**Mechanisms of action**	**Examples**
**Increased burden of disease**	Migration leads to the migration of infectious diseases infecting new non-immune hosts.	The United Nations High Commissioner for Refugees (UNHCR) estimates that there are 15.3 million refugees and a further 26 million internally displaced persons (IDPs) worldwide [116]. Migration in camps that are overcrowded leads to situations where sanitation is not adequate and outbreaks can occur [117], a consideration going back nearly a century to the start of World War II [118].
	Breakdown of prevention programs leading to an increase in vector-borne diseases, such as malaria and trypanosomiasis.	Afghanistan has seen an increase in malaria after it had successfully controlled this disease in the 1970s and the Democratic Republic of Congo has had a rise in trypanosomiasis in conjunction with the rise in conflict [119]. Refugee camps in Sierra Leone and Guinea have both seen outbreaks of Lassa fever from the infestation of rodents [120,121].
**Susceptibility of the population to infectious diseases**	Reduced immunity from malnutrition, inadequate coverage of immunizations and the loss of herd immunity and the lack of innate immunity to unseen infective organisms.	Afghan refugees from a malaria-free region who fled to Pakistan in 1981 had a prevalence of malaria more than double that of the local population, and a ten-fold increase in burden over the following decade [122].
**Breakdown of the healthcare system**	Healthcare may be suspended or diminished [2] and funds diverted from it to armed forces or security actors. This leads to reduced detection and treatment of infectious diseases and potentially to increased rates of antibiotic resistance. This is combined with difficulties in accessing the services that do function due to fear of movement or breakdown of the transport networks.	The restriction of transport networks by the Maoist rebels in Nepal in 2005 held up the supply of vaccines, vitamin A, and deworming drugs to nearly 3.6 million Nepalese children [123].
		Even normally functioning immunization programs can be affected by security concerns, the polio eradication program in Afghanistan being an example [124]. During the conflict in Bosnia and Herzegovina in the early 1990s, immunization rates fell from approximately 95% pre-conflict to around 30% [125]. Health facilities may themselves come under deliberate attack [8][124,126]. In the Nicaraguan conflict of the mid-1980s, approximately a quarter of the health facilities were partially or completely destroyed [54].
	Movement of healthcare workers	Healthcare workers often have the socioeconomic wherewithal to migrate during conflicts. A report from the International Committee of the Red Cross quotes an Iraqi Ministry of Health estimate that 18,000 of the country’s 34,000 doctors left [127]. Liberia is thought to have seen a decrease from 237 to 20 doctors during recent conflict [128].

Sexual violence against women has been mentioned, and sexually transmitted infection rates may rise in conflict settings, as seen with syphilis and gonorrhea in the Second World War [129]. Increased infections are especially common in refugee or IDP populations [130], and in situations characterized by alcohol use and riskier sexual practices [75]. They have also been linked with psychiatric conditions and psychosocial factors. In war-affected Eastern Uganda, major depressive disorder and sexual torture were associated with high-risk sexual activities, which were themselves associated with HIV transmission [131]. It is thought that the conflict in Mozambique led to higher rates of syphilis in pregnant women, some of whom experienced sexual abuse and repeated rape while being held by the insurgents [132]. HIV rates may also be augmented by untreated sexually transmitted infections, lack of condoms, increased incidence of sex work and lack of health education [80,119]. An estimated 70% of Rwanda’s rape survivors were infected with HIV [133].

#### Intergenerational associations

Infectious diseases can affect subsequent generations through direct effects on pregnant women or through the effects of long-term morbidity on their future health, reproductive capacity and finances. The Spanish influenza outbreak, which resulted in up to 40 million deaths, is considered to have originated in and been exacerbated by the conditions of the First World War [134,135], and research on prenatal exposure suggests both long-term health costs, such as increased rates of cardiovascular disease and potentially increased disability, and economic penalties [136,137]. More specifically, infection *in utero* may lead to abortion or congenital malformations, and many infections can trigger premature birth. A fall in immunization rates may be accompanied by reductions in herd immunity: outbreaks of rubella, which can lead to severe congenital abnormalities, occurred during the conflict in Bosnia and Herzegovina [138]. Congenital infections lead to impaired cognitive development, respiratory and gastrointestinal disease, and may leave children vulnerable to infections during their lifetime [139].

Infections such as HIV and syphilis can be transmitted vertically from mother to child, causing acute infections, fetal or infant death or chronic childhood conditions. A review of neurodevelopmental outcomes showed that vertical transmission of HIV was associated with poorer neurodevelopmental outcomes in children from resource-poor countries [140]. Likewise, repeated malarial infections and continuing parasitemia are associated with reduced cognitive ability in children in the short- and long-term, with effects on their future economic capacity [141-143].

Lack of human resources adversely affects any future health system. Conflict-driven collapse of the education system, for example, reduces the pool of people who could become health workers. The best-known example is the breakdown of the education system under Cambodia’s Khmer Rouge regime, in which teachers were systematically imprisoned or executed. This not only affected the education of children at the time, but also led to a longer-term loss of knowledge and skills from society.

As common users of healthcare services, pregnant women are especially vulnerable to healthcare service disruptions, leading to the exacerbation of illness. An example of this would be the disruption to the supply of drugs and other medical equipment, as described in northern Sri Lanka during the recent civil war. Obstetric complications, such as pre-eclampsia, were common, potentially contributing to the higher rate of low birthweight that was found [144]. In Palestine, military checkpoints delaying access to maternity facilities were associated with an increase in home births from 8% in 1999 to 33% in 2002 [145], while in Nepal’s recent civil war, disruption of a hospital’s electricity supply led to Caesarean sections being performed by torchlight [123]. More generally, maternal mortality rises during conflict. About half the countries suffering from conflict have maternal mortality ratios >200 per 100,000 live births [8]. The maternal mortality ratio during the conflict in Bosnia and Herzegovina was approximately 75% higher than in the periods immediately before and after [146]. A catastrophic event in its own right, maternal mortality also has damaging effects on the remaining family members, particularly children. Evidence from a non-conflict area of Bangladesh shows very high mortality among younger children - up to 70% for a surviving neonate within two years of the maternal death - approximately 10 times higher than if neither parent had died. Higher mortality persists in children five- to ten-years old, for whom the probability increases five-fold [147]. Similar evidence from Haiti shows increased odds of mortality after a maternal death of 55% for children younger than 12-years-old [148].

### Nutrition

#### *Maternal exposure*

Malnutrition is common in conflicts and is predominantly manifest in children [149]. During a recent famine in the Horn of Africa, rates of global and severe acute malnutrition reached levels of 50% and 35%, respectively [150]. Food security falls during conflict for reasons ranging from reduced crop production to breaks in the supply chain and trade restrictions [9,151]. Food resources may be destroyed deliberately as a means of harm or population control: in 1980, 140,000 hectares were destroyed by Ethiopian government forces to prevent their use by rebel groups [152]. The diversion of resources away from healthcare and food supply to military expenditure in war can adversely affect population health. Despite an increase in overall gross development product per capita, the early Nazi regime saw an increase in mortality and a decline in child height [153]. International sanctions, a favored government response to civil conflict, may generate similar effects. Neonatal mortality, for example, increased substantially in Iraq during the 1990s [154].

There is some evidence that breastfeeding rates can decline in war times and the adverse effects of this can be greater. In the war in Croatia in the early 1990s, breast-feeding duration was found to be shorter in affected areas [155]. In Guinea Bissau in the late 1990s, infants who were weaned earlier during war periods had higher mortality rates than breastfed infants compared to the pre-war years where this difference was not present [156]. In addition to not having breast milk, we would postulate that disruption to formula milk supplies and unclean water would compound the adverse effects. It was shown that children who were never breastfed were more likely to be malnourished [157].

War may contribute to either acute malnutrition and increased mortality or chronic malnutrition leading to stunting and subtle prolonged deficits associated with lower school attainment and reduced adult income [158]. Nutritional deficiencies may be in macronutrients, providing the basic energy and substrate for growth, or in micronutrients, including vitamins and minerals that promote cellular function. Micronutrient deficiency diseases are common in conflicts: scurvy in Afghanistan from vitamin C deficiency [159], pellagra in Angola from niacin deficiency [160].

#### *Intergenerational associations*

Malnourished mothers pass on the stress to their children, whose poor nutritional status may affect subsequent generations. In the Dutch Hunger Winter of 1944 to 1945 during the German occupation, maternal under-nutrition was associated with increased risk of low birth weight [161]. Although not directly attributed to malnutrition, a higher proportion of babies were born with moderate low birth weight in Croatia during the war period (1991 to 1995) [162]. Low birth weight is the strongest risk factor for mortality in early infancy [163] and is associated with reduced educational attainment and physical work capacity [158]. Subtler effects include maternal micronutrient deficiencies, such as iron deficiency anemia [164], and conditions that directly affect the fetus, such as neural tube defects associated with folate deficiency [165]. Prenatal malnutrition, leading to low birth weight, results in lower subsequent height and lean mass [166]. Short maternal stature is associated with an increased risk of gestational diabetes, macrosomia, and birth injury and shorter gestation [167-169]. Among Hmong refugees from the Second Indochina war, children displaced during infancy were shorter as adults, whereas children born during the war were found to have greater adiposity, particularly central adiposity [170]. Such growth penalties may take generations to resolve. Maternal short stature is a risk factor for obstructed labor, Caesarean section and low birth weight [171-173], potentially generating a long-term intergenerational cycle [15]. Contrasting with sperm production in men, women’s ova are formed primarily early in life, and damage to the reproductive system in young girls, from infections, trauma or harmful substances, for example, can have long-term effects on their reproductive capacity and their children [174,175].

Peri-conceptional, fetal and infant malnutrition can affect the risk of non-communicable disease. People exposed to maternal malnutrition *in utero* during the Dutch Hunger Winter showed increased risk of obesity, hypertension, cardiovascular disease and type 2 diabetes [176-178]. The longer-term effects depended on when in gestation the lack of nutrition occurred and the period for which there was a deficit, and there is some evidence for a difference in the long-term effects between male and female offspring [179,180]. Although the evidence is conflicting, it may be that the detrimental effects of the Dutch famine extended into the following generation, with the offspring of women born in the famine found to have a lower birth weight [161,181]. Similar associations between early-life famine exposure and subsequent elevated chronic disease risk were reported following the Biafran conflict of 1968 to 1970 [182], and the Chinese famine of 1959 to 1961 [183-186]. In the latter, early-life exposure was also associated with increased risk of schizophrenia [187,188]. In non-conflict settings it is common for chronic disease in adults to affect their offspring, both biologically and economically via loss of earnings. An example of this would be the propensity to obesity and the development of gestational diabetes leading to preterm birth and macrosomia.

Deliberate military efforts may not only impair the capacity for food production but also increase exposure to toxins. For example, bombing and Agent Orange were used in Vietnam to ‘deny the enemy sources of food and means of cover’ [189]. Approximately 11 to 12 million gallons of Agent Orange were sprayed over nearly 10% of South Vietnam between 1961 and 1971. While research on intergenerational effects continues, the WHO describes dioxin, the active element of Agent Orange, as ‘highly toxic’ and able to cause ‘reproductive and developmental problems’ [190,191].

### Multiplicative effects

**Figure 3:**
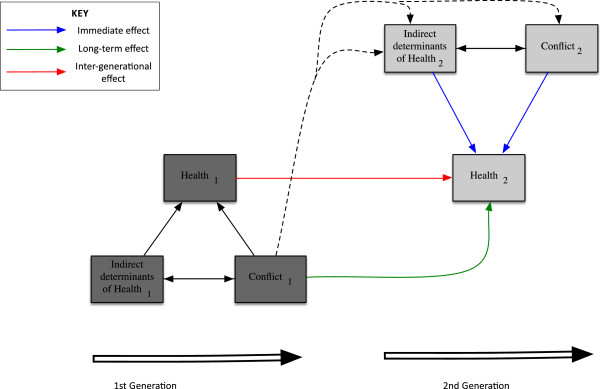
Schematic diagram showing how both current and past events affect the health of children across multiple generations.

The intergenerational harms of short conflicts are mitigated by post-conflict recovery of economies, societies and health and schooling systems. In long-lasting conflicts, however, they are exacerbated by on-going fighting to produce a composite effect whereby the immediate effects of conflict combine with long-term and intergenerational effects, as illustrated schematically in Figure 3.

It is difficult to provide definitive evidence for this as prior susceptibility is hard to quantify, but we propose two illustrative scenarios. In the first, an increase in preterm births, perhaps associated with an increase in congenital infections, makes children more susceptible to subsequent infection. This susceptibility is exacerbated by the increased wartime burden of disease. Infection exacerbates wartime under-nutrition, which combines with reduction in the food supply in a downward spiral that reduces a child’s capacity to cope with physical insults. These multiple deficits occur within a health system that already cannot cope. In the second scenario, children growing up in a long-lasting conflict region suffer from the intergenerational effects of mental ill-health, as well as an ongoing conflict stress burden. For example, a child inherits an impaired cortisol response through parental stressful experiences, and this makes it harder to cope with the subsequent stressful events the child faces herself.

## Discussion

We have given examples of how conflict can have long-lasting intergenerational effects, working through parental exposure to violence, mental health stressors, infection and nutrition (summarized in Table 2). Much has been written about the health effects of war but the literature on its enduring effects is sparse. With our growing understanding of the developmental origins of health and disease, it is becoming evident that the extreme conditions that conflict imposes can have effects that last for generations, making a ‘brief’ conflict a misnomer. Many interrelated pathways have been identified between parental exposure and subsequent generations, but further evidence is required to estimate the magnitude of their effects. Given the range of conflict scenarios, there is likely to be a great deal of heterogeneity.

**Table 2:** Summary of maternal and intergenerational effects

**Key areas**	**Maternal exposure - possible consequences**	**Intergenerational associations - increased risk of**
Violence	Domestic violence	Congenital malformations
	Mental illness/physical trauma	Low birth weight
	Rape	Perinatal morbidity and mortality
	Trafficking and prostitution	Premature birth
		Altered physical and mental development
		Neglect and abuse
Mental health	PTSD	Impaired growth
	Depression	Poor educational attainment
	Anxiety	Neuroendocrine and immune system modulation
	Psychosomatic disorders	Infection
	Drug and alcohol abuse	Mental illness
		Higher suicide rates
		Social functioning impairment
Infectious diseases and health systems	Increased transmission of infectious diseases	Fetal or infant mortality
	Malnutrition	Disability
	Obstetric complications	Cardiovascular disease
	Increase in maternal mortality	Congenital malformations
		Impaired cognitive development
Nutrition	Malnutrition including micronutrient deficiencies	Neonatal mortality
	Obstetric complications	Low birth weight
	Maternal mortality	Malnutrition/under-nutrition
		Impaired growth and development
		Educational under-attainment
		Congenital abnormalities
		Non-communicable diseases
		Future reproductive capacity

Our categorization of potential exposures and effects is illustrative and artificial in the sense that their interactions are complex. Physiological and pathological effects of stressors – trauma, infection, malnutrition – are linked in a complex system with institutional factors, such as education and health system challenges, and social factors, such as cultures of alcohol use and violence. Prior conflict creates a population with a reduced capacity to cope with adversity, and ongoing or subsequent conflict may compound this and exacerbate the adverse health effects. The prevention of conflict and its effects on health are crucially important public health concerns [11], but the intergenerational picture has received little attention so far. In many cases, the causal link between conflict and intergenerational outcomes is lacking, and the paucity of research reflects at least partly the challenges of working in current or recent conflict zones. Where evidence from conflict situations is limited, we have hypothesized on the basis of available evidence or discussed evidence from non-conflict situations.

### Intergenerational justice

Similar to the growing calls for consideration of future generations in climate change debates, we call for policy makers to consider them with respect to conflict. The notion of intergenerational justice emphasizes a temporal dimension, giving future generations rights that those currently alive should maintain. The extent to which existing legislative powers can be used to uphold these rights is uncertain, but legal bodies could potentially extend their scope to the violation of the rights of future beings. Statutes such as the UN Convention on the Rights of the Child (Articles 38 and 39) already exist to protect the rights of children in war [192]. The arguments we lay out are aligned with gender-based social justice since in many parts of the world women receive fewer resources in general and particularly in terms of access to healthcare [193]. The idea of intergenerational justice can also be aligned with a present day rights-based approach to help protect not only the current population but also future generations. In this sense, we do not see a clash between the two.

It is not within the scope of this paper to adequately review the interventions that may be of benefit in preventing the future adverse effects of war, but we have tried to summarize the main categories in Box 2. Many of these are the same as those required for the current population, but the imperative to protect certain groups, such as pregnant women, is reinforced. Past conflicts may have led to positive changes, such as the removal of oppressive regimes, as well as negative impacts. However, our discussion highlights the importance of minimizing the likelihood of conflict when seeking such positive changes. Interventions to break the cycle of transmission should be examined at the point at which governing bodies and non-state actors are considering going to war, and in its aftermath, as well as during conflict. In examining the health of a population, previous insults need to be considered in order to understand fully the situation and to initiate solutions. Importantly, policy makers should bear in mind that a population may take multiple generations for the adverse health effects of conflict to be negated as a region attempts to return to its premorbid state or moves on to a new post-conflict one and it is possible that a return to the previous state may never happen if conflict changes the status quo within a given area. Conflict-related public health interventions need to be sustained for a number of years and adapted over time to cope with changing needs.

## Summary

In this article, we summarize how the effects of war can propagate across generations. We hope this review will stimulate debate and research on the long-term and intergenerational health effects of conflict and their mechanisms and contribute to the discussion of the costs of war. The evidence we have included strengthens the position that violent conflict should be avoided and indicates that intergenerational effects should be included routinely in the anticipated and estimated consequences of war.

## Endnote

^a^WHO definition of conflict: ‘The instrumental use of [armed] violence by people who identify themselves as members of a group – whether this group is transitory or has a more permanent identity – against another group or set of individuals, in order to achieve political, economic or social objectives’. Wars are armed conflicts with more than 1,000 battle-related deaths in any one year.

### Box 1: Biological effects of PTSD

Increased stress levels in mothers can act in a similar fashion to under-nutrition, potentially mediated by changes in the hypothalamic-pituitary-adrenal (HPA) axis [194]. Occurrence of PTSD is thought to be influenced by epigenetic changes, with possibly both genome-wide and specific changes in genes such as *DLGP2 *[195]. Exposure to massive stress during pregnancy has been associated with epigenetic programming of the HPA axis *in utero*, leading to an increased susceptibility to mental illness in the child [90]. The mechanism by which this is believed to occur is that traumatic events lead to epigenetic changes to the glucocorticoid *GR* gene that subsequently alter offspring cortisol response to future events. Radtke *et al*. showed lasting effects of the methylation of glucocorticoid receptor genes associated with intimate partner violence against the mother around the time of pregnancy [91]. These findings from research on humans are supported by animal studies. For example, expression of the glucocorticoid receptor gene in the rodent hippocampus is modulated by the level of care received by the mother [196,197]. Further to this, the administration of drugs that alter DNA methylation can reverse altered behavior, as shown by the use of the demethylating substance trichostatin and methylating substance methionine [198,199].

### Box 2: Using the evidence of intergenerational consequences of conflict: some possible strategies

Prevent the onset of war and violent conflict

When conflict starts, all options to reach an alternative peaceful solution have rarely been exhausted [200]. There may be strong political or economic motivations behind the decision to go to war or resort to violent conflict. These may include the need to do so for ‘humanitarian’ reasons, although recent military action initiated on this basis has caused such a degree of mortality, morbidity and destruction that the humanitarian reason has been rather discredited. However, there is potentially a case to be made for going to war to prevent death, injury and destruction.

Viewed objectively, there will be a range of reasons for going to war or taking violent action and a range of reasons for not doing so; evidence of the health consequences of taking violent action will fall within the latter. The greater this evidence is, the harder it will be to justify war. Intergenerational consequences for health are an under-researched result of war that could tip the balance against it, help put a brake on the push to war and ensure that other peaceful options are pursued as a priority.

Factor in a longer-term perspective if contemplating going to war

The link between the likely longer-term health consequences of going to war and the conflict itself can be hard to establish definitively. The more time passes, the more possible confounders have to be taken into account, making direct causal links difficult to prove. However, combining disciplines and research to illustrate these links in the area of child health can be compelling, as there is a general consensus that children should be protected during war and that future generations are innocent. This makes a growing body of evidence that supports the link between conflict and future health problems for children a strong advocacy tool for conflict prevention.

Ensure a long-term component to interventions both during and after conflict, taking long term consequences into account

The type of interventions used to meet health needs caused by conflict, and the resources that are dedicated to them, should be decided on the basis of need. Much work has been done on how those needs can be accurately assessed [201] and minimum standards of response assured [202]. If need can be shown to be more long-lasting and serious than previously thought, particularly in relation to mother and child health, it could influence needs assessments, minimum standards and intervention planning. This would also help make the case for sufficient and appropriate resource allocation.

Ensure extra care is provided for vulnerable groups, particularly pregnant women

Evidence of the intergenerational health consequences of violent conflict will reinforce the degree of vulnerability of pregnant women to health shocks. Pregnant and lactating women are already recognized as a priority group for humanitarian health programs [203], but there is still much to be done before programs are fully integrated, effective and adequately resourced. A better understanding of the consequences of not having optimal and well-resourced programs will be a strong advocacy tool to use with donors and others.

Upholding international conventions to safeguard children through intergenerational justice

International law that relates to the rights of children is almost universally recognized and key parts of it are considered to be customary law [204]. Better established links between violent conflict and the health and wellbeing of children not yet born could contribute to holding those who initiate war or violent conflict to account for the consequences of their actions. This, in turn, would be a further restraining factor on initiating violent conflict.

## Abbreviations

CI: confidence interval; HPA: hypothalamic-pituitary-adrenal; IDP: internally displaced person; OR: odds ratio; PTSD: post-traumatic stress disorder; UNHCR: United Nations High Commissioner for Refugees.

## Competing interests

The authors declare that they have no competing interests.

## Authors’ contributions

DD reviewed the literature and wrote the initial draft. MB, DO, ES, and JW critically reviewed and amended it. All authors read and approved the final manuscript.

## Supplementary Material

Additional file 1 Supporting material: literature review method. Click here for file
