# Supplementary material for: The intergenerational effects of war on the health of children
Source: BMC Med. 2014 Apr 2;12:57. doi: 10.1186/1741-7015-12-57 (PMC3997818; doi:10.1186/1741-7015-12-57)
Supplement: Additional file 1 — Supporting material: literature review method. [file 1741-7015-12-57-S1.docx]

**Additional file 1**

**Supporting material: Literature review method**

The literature search was performed using the Medline and Psychinfo databases. The titles/abstracts were screened for relevance of the effects of mass conflict or war on health in the next generation. From the literature searches, twenty articles (Figure 1a = 6, Figure 1b = 14) were included in the paper. We then considered the reference lists or suggested articles for further evidence, from which 130 articles were included. This was supplemented by additional searches of the literature using Medline, Psychinfo and Google Scholar as specific questions arose.

**Figure 1a Primary literature search**

**Figure 1b Literature search: background material**
